# Variations in social care need reporting amongst GP practices in England: a retrospective cohort study in people with multimorbidity

**DOI:** 10.1186/s12875-025-03107-2

**Published:** 2025-11-27

**Authors:** Dan Burns, Glenn Simpson, Zlatko Zlatev, Lucy Smith, Jakub Dylag, Miriam Santer, Michael Boniface, Andrew Farmer, Hajira Dambha-Miller

**Affiliations:** 1 Digital Health and Biomedical Engineering, ECS, University of Southampton, Southampton, UK; 2 Primary Care Research Centre, University of Southampton, Southampton, UK; 3 Nuffield Department of Primary Care Health Sciences, University of Oxford, Oxford, UK

**Keywords:** Multimorbidity, Primary care, Clinical Practice Research Datalink (CPRD),Social care needs, Electronic health records

## Abstract

**Background:**

Multimorbidity, the presence of multiple chronic health conditions, presents significant challenges in both health and social care settings. Addressing social care needs, such as assistance with daily activities and support for managing finances, is crucial in care management patients with multimorbidity. However, variability in the documentation and reporting of these needs remains poorly understood. This study aimed to quantify the variations in social care need (SCN) reporting across GP practices in England.

**Methods:**

We conducted a population-based study using electronic health records from a national sample of 873,092 individuals with multimorbidity. Inclusion and exclusion criteria were applied to determine the final cohort, with demographic and clinical data extracted. We analysed SCN reporting rates at the practice level, using interquartile ranges (IQRs) and intra-class coefficients (ICCs) to assess variability. Factors influencing SCN reporting were examined, including long-term conditions, demographic variables, and socio-economic deprivation.

**Results:**

Significant variability was observed in SCN reporting across GP practices. Outcomes related to mobility and residential needs showed the greatest differences in reporting rates. Moderate correlations were observed between certain SCN categories, such as mobility and activities of daily living, as well as disability and financial needs. Patients with long-term conditions, such as dementia and multiple sclerosis, were more likely to have their SCNs reported, while other multimorbidity conditions showed lower reporting rates. Demographic factors, including gender and socio-economic deprivation, were associated with higher reporting rates, particularly for females and patients in more deprived areas.

**Conclusions:**

This study highlights the significant variability in the documentation of social care needs across healthcare practices, using electronic health records in a large population-based sample. The findings emphasise the need for standardised reporting practices to ensure comprehensive care for individuals with multimorbidity, particularly those from more deprived socio-economic backgrounds and with complex care needs. Improved reporting could enhance care coordination and reduce health inequalities.

## Introduction

 Multimorbidity, which is commonly understood as the presence of two or more chronic health conditions, poses significant and growing challenges to health and social care systems across the globe [1–3]. Effectively managing patients with multimorbidity requires a holistic approach that integrates both clinical and social care needs [4, 5]. Social care needs, such as assistance with daily activities, access to community resources, and support in managing finances, are experienced by up to 50% of multimorbidity patients [6]. Research in England has shown that an increased prevalence of multimorbidity is directly linked to increased local authority expenditure on social care [7]. There is an interplay between multimorbidity and social care needs, which play a crucial role in determining health outcomes for individuals with multimorbidity [8, 9]. For instance, a study in New Zealand [10] found that multimorbidity patients had at least one social need, and a UK study [11] showed that difficulties with activities of daily living are predictive of nursing home admission. Unmet social care needs can worsen difficulties associated with chronic conditions, resulting in poorer health outcomes and increased healthcare utilisation [12]. For instance, multimorbidity can be a barrier to securing employment and access to a reliable income and individuals with financial needs may face barriers to accessing essential support services, further complicating their medical management [13]. Further, a survey of 200,000 patients with multimorbidity, found less than half felt their needs were met by local services, and this reduced to less than a third for individuals with five or more conditions, declining further among ethnic minorities, the socially isolated, the frail and those needing support with activities of daily living [14]. Therefore, addressing social care needs is vital for delivering comprehensive and holistic care and promoting better health trajectories and outcomes for patients with multiple long-term conditions [12].

As primary care increasingly focuses on these social care dimensions, understanding how social care needs are reported within primary care settings becomes critical [15]. For example, effective reporting can highlight good practices and identify opportunities to enhance patient care and health service delivery [16, 17]. Research has estimated that approximately one-fifth of GP consultations are the result of social needs (e.g., financial management difficulties, poor housing conditions), although the systematic documentation and reporting of these needs remain poorly understood [18]. This may reflect substantial variability in care provision across practices, where some patients receive thorough assessments of their social care needs and appropriate care interventions, whilst others may go unrecognised and have unmet social care needs. Additionally, the lack of reporting and understanding of social care needs may impede the development of effective interventions that address both clinical and social aspects of patient care [19, 20]. Furthermore, underreporting social care needs can lead to missed opportunities for social prescribing and inadequate support for patients navigating complex health and social care systems [21]. Inadequate documentation compromises individual patient care and also undermines public health initiatives aimed at reducing health inequalities and addressing social determinants of health [21]. Understanding these variations may offer opportunities for enhancing care coordination, informing policy, and ultimately improving quality of life for patients with multimorbidity by enhancing holistic care plans that consider both clinical and social care needs [4, 5]. In this study, we have quantified variations in reporting of social care needs amongst GP practices in England.

## Methods

### Study design and population

We conducted a retrospective cohort study using data from the Clinical Practice Research Datalink (CPRD) (GOLD), focusing on adults aged 18 years and older with multimorbidity registered with a General Practice (GP) in England. The study population included patients registered at any point between January 1, 1987, and December 31, 2020. The CPRD database provides a population-based sample representative of England in terms of age, sex, and ethnicity, comprising over 59 million unique patients, with more than 16 million currently registered [22, 23].

### Curation of data

The dataset included only those individuals who had a record of at least two long-term conditions at any time point during their registration. Social care needs reporting was defined through eight specific domains: activities of daily living (ADL), mobility needs, financial needs, disability needs, community care needs, residential status needs, social networking needs, and bereavement needs [13] (Table 1). The details of these domains, including the codes, have been described in detail in another publication [24]. In this study, ‘financial needs’ were defined according to CPRD coding, which captures support with managing finances as part of instrumental activities of daily living (IADLs). This differs from the broader concept of financial difficulties or welfare needs (e.g., benefit applications, income support, or general financial hardship), which fall outside the scope of CPRD coding. Our use of the term therefore reflects social care–related financial needs rather than broader welfare considerations. In CPRD, information is recorded using clinical and administrative codes, which are standardised terms used by general practices to capture patient characteristics, diagnoses, treatments, and other relevant factors. These codes form the basis for identifying and analysing social care needs in this study. A social care need was considered if a corresponding code was identified while the patient was registered at the current practice. We extracted indicators of social care needs to assess the extent of reporting across the specified domains.

**Table 1:** Domains and descriptions of social care needs. This table defines and describes all aspects of social care needs in each of the eight domains. Relevant codes for these aspects were identified and utilised in the analysis using clinical practice research datalink (CPRD) data

Domain	Description
1. Activities of daily living needs	Social care needs associated with carrying out activities of daily living (ADLs) including: • Ambulating - the extent of an individual’s capability to move from one position to another, perform basic movements and dexterity. • Feeding - ability to feed oneself without assistance. • Dressing - ability to put on their clothes and select appropriate clothing. • Personal hygiene - ability to undertake basic washing, bathing, and grooming, maintain personal hygiene including dental, nail, and hair care. • Continence – ability to control bladder and/or bowel function. • Toileting - ability to get to and from the toilet, using it appropriately and cleaning oneself.
2. Mobility needs	Social care needs resulting from difficulties with physical mobility, require the provision of mobility aids or equipment, professional input and care and financial support with mobility including: • Wheelchair use. • Non-wheelchair mobility aid use (e.g., walking stick, Zimmer frame, mobility scooter). • Professional care staff provision to assist with mobility. • Receives disability living allowance. • Receives mobility allowance.
3. Financial needs	Social care needs relating to managing finances and associated employment-related difficulties, and receiving assistance from a range of financial support services, including: • Difficulty budgeting and handling money. • Employment status (e.g., unemployed, or long-term sick). • Referral and/or input from a financial adviser or financial service providers. • Receipt of assistance from support services such as food banks and accessing the affordable warm programme. • Difficulty writing cheques, using a credit cards, carrying out arithmetic reasoning related to money and difficulty managing bank accounts.
4. Disability needs	Social care needs resulting from specific disabilities or impairments including: • Mental health disability. • Intellectual disability. • Learning disability. • Speech, hearing, and sensory disabilities. • Any state assessment of disability.
5. Community care needs	Social care needs requiring support from a range of community health and social care services and practitioners, including: • Community physiotherapist or occupational therapy. • Drug and alcohol team. • Mental health team. • Social care services. • Outreach or voluntary services. • Community nurses, specialist nurses or matrons. • Community secondary care professionals. • Audiology. • Palliative care team. • Dietician. • Community care navigator or social care prescribers. • Pharmacy input.
6. Residency status needs	Social care needs relating to residential status including: • Hospice care. • Nursing home care. • Care home. • Own home with adaptations.
7. Social care networking needs	Social care needs associated with an individual’s ability to socially connect, network, and participate in social care activities including: • Ability to maintain meaningful relationships with family, friends, and others. • Ability to socialise and mix with others in social care contexts. • Ability to effectively communicate verbally and non-verbally. • Ability to participate in hobbies, leisure, and community activities. • Access to and ability to organise transportation. • Input from professional services to socially connect and participate such as day units or visits from charities for loneliness.
8. Bereavement needs	Social care needs arising from family bereavement including: • Death of a partner, husband, wife, sibling, child including neonatal and postnatal death, sudden infant death, maternal death. • Input from professional services including referral to or use of bereavement counselling or therapies.

To define multimorbidity, we use the presence of two or more long-term conditions using both Read/SNOMED codes and Hospital Episode Statistics (HES) (ICD-10 codes), with the date of the earliest identification extracted using a comprehensive list of 56 long-term conditions that was agreed through earlier national consensus with stakeholders and researchers across the United Kingdom [20]. A detailed description of these conditions and the corresponding data codes can be found in our previously published work [20]. We also extracted demographic information, including age, sex, ethnicity, and the patient’s Index of Multiple Deprivation (IMD) quintile.

### Statistical analysis

Descriptive statistics were used to review the overall reporting rates of social care needs (SCN) at the patient level, comparing those with reported needs to those without. At the practice level, we calculated the reporting rate for the following outcomes: 1) any SCN reported and 2) each individual SCN class: ADL, Disability, Community, Mobility, Residential, Social Network, Bereavement, and Finance. We reported the range, median, and interquartile range (IQR) for these metrics.

Multi-level logistic regression analysis was performed to model the probability of any SCN being reported and each SCN class. Patient-level exposures included age (modelled as a continuous variable), gender, ethnicity, patient IMD, and any history of the 59 long-term conditions in our conditions set. Practices were included as a random effect, excluding those with zero events from the analysis. We calculated the intraclass correlation coefficient for each model and investigated the odds ratios associated with the patient-level covariates. Additionally, we explored the correlation between SCN classes at the practice level using scatter plot visualisations and computed Spearman’s rank correlation for each pair of SCN classes.

To select our cohort, we excluded patients who met the following criteria: 1) no multiple long-term conditions, 2) patient transfers out of the practice before December 31, 2019, 3) patient registrations at the practice after December 31, 2019, 4) patient deaths before December 31, 2019, and 5) incomplete demographic data (i.e., unknown gender, ethnicity, or IMD). For each specific SCN, we included only practices with at least one reported event. At the patient level, we removed patients for whom the particular social care need was reported before practice registration.

## Results

### Study population

**Fig. 1:**
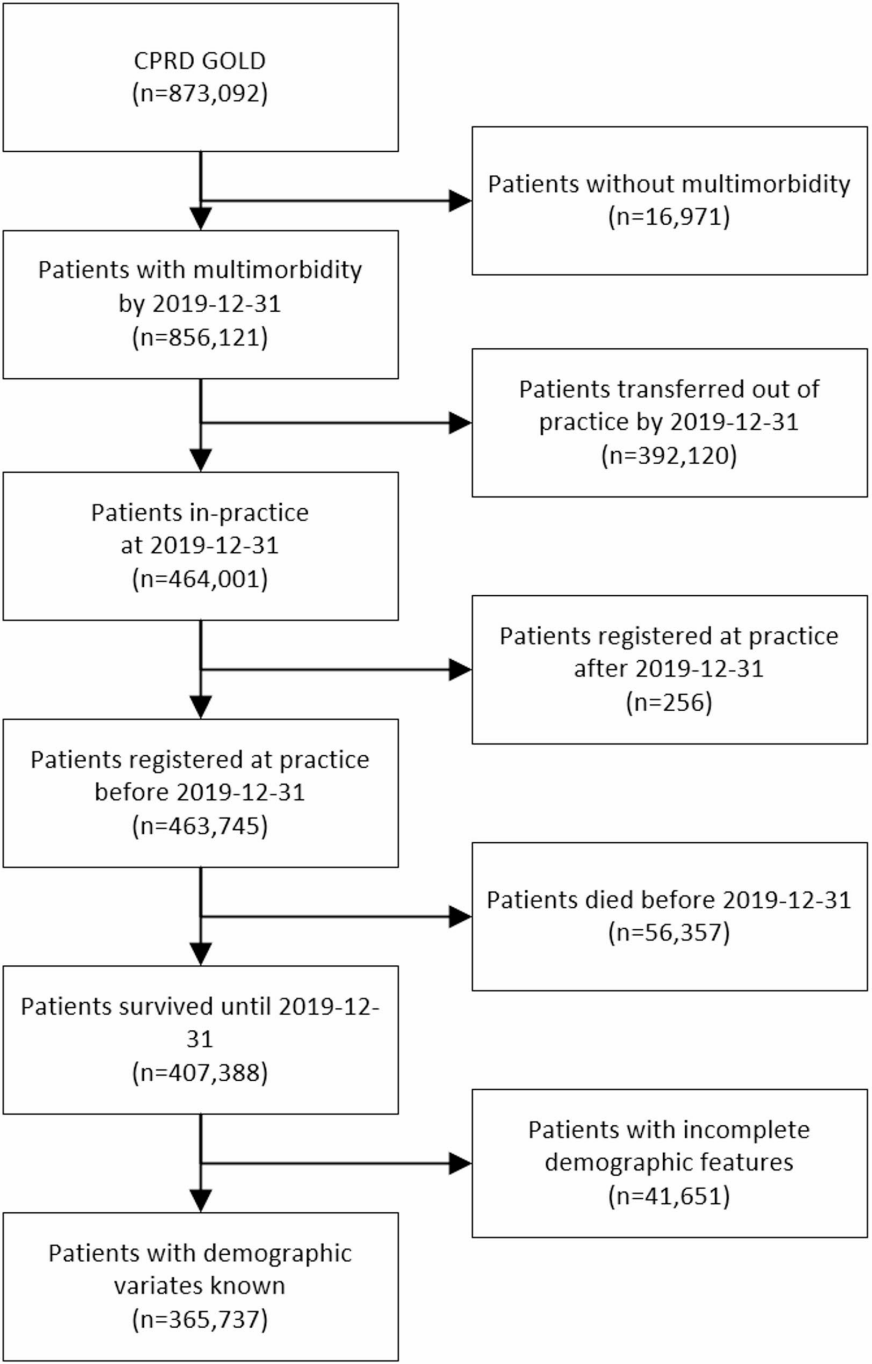
Cohort selection diagram

The cohort selection diagram is presented in Fig. 1, with a detailed description of the final cohort provided in Table 2. Initially, the dataset contained 873,092 individuals with multimorbidity. Following the application of inclusion and exclusion criteria, 16,971 individuals with only a single condition or no conditions at the study date were excluded. Additionally, 392,120 individuals who had transferred out of the practice by the study date were removed, alongside 256 patients who registered after the study date. Of the remaining cohort, 56,357 individuals had passed away before the study date. Finally, 41,651 patients were excluded due to incomplete demographic information.

**Table 2:** Description of cohort demographics stratified by whether a SCN was reported. Entries represent the count of patients in each category, with the percentage representing the proportion of patients in that category with respect to the outcome group

Variable	Category	No SCN reported (*N* = 248060)	SCN reported (*N* = 117677)
Age	18–24	2,412 (1.0%)	591 (0.5%)
	25–34	13,479 (5.4%)	3,572 (3.0%)
	35–44	21,550 (8.7%)	6,457 (5.5%)
	45–54	36,155 (14.6%)	13,459 (11.4%)
	55–64	49,312 (19.9%)	21,321 (18.1%)
	65–74	56,748 (22.9%)	26,806 (22.8%)
	75–84	46,612 (18.8%)	26,762 (22.7%)
	85+	21,792 (8.8%)	18,709 (15.9%)
Sex	Male	108,181 (43.6%)	46,771 (39.7%)
	Female	139,879 (56.4%)	70,906 (60.3%)
Ethnicity	Asian	7,573 (3.1%)	4,095 (3.5%)
	Black	3,856 (1.6%)	2,127 (1.8%)
	Mixed	1,324 (0.5%)	602 (0.5%)
	Other	3,225 (1.3%)	1,515 (1.3%)
	White	232,082 (93.6%)	109,338 (92.9%)
Patient Index of Multiple Deprivation Quintile (1st is least deprived)	1st	48,472 (19.5%)	20,688 (17.6%)
	2nd	50,042 (20.2%)	22,236 (18.9%)
	3rd	54,804 (22.1%)	26,151 (22.2%)
	4th	50,856 (20.5%)	24,776 (21.1%)
	5th	43,886 (17.7%)	23,826 (20.2%)

Table 2 presents a contingency table displaying the distribution of age group, gender, ethnicity, and Index of Multiple Deprivation (IMD) quintiles for patients with a Social Care Needs (SCN) report versus those without. The two groups exhibit similar characteristics in terms of sex and ethnicity, with both populations predominantly comprising White individuals and a higher proportion of females compared to males. There is a limited difference in the age distribution, with the SCN-reported population tending to be older, suggesting that age is a factor associated with the reporting of a SCN. The IMD quintile distribution also shows a shift, with fewer individuals in the least deprived quintiles and a higher proportion in the more deprived quintiles among those with SCN reports.

### Variation in social care need reporting at practice level

Considerable variability in SCN reporting rates was observed across practices, as illustrated in Table 3. Extreme differences were found between the interquartile range (IQR) and the overall range for all outcomes considered. The outcomes exhibiting the most substantial variations were mobility (IQR 1–2%; range 0–32%) and residential needs (IQR 0–1%; range 0–40%).

**Table 3:** Practice-level description of reporting rates

	Any SCN	ADL	Disability	Community	Mobility	Residential	Social Network	Bereavement	Finance
SCN reporting rate, median (IQR)	30% (21% − 40%)	5% (2% − 8%)	1% (1% − 3%)	20% (11% − 31%)	1% (1% − 2%)	0% (0% − 1%)	0% (0% − 1%)	2% (1% − 4%)	2% (1% − 4%)
SCN reporting rate, range	0% − 60%	0% − 22%	0% − 24%	0% − 57%	0% − 32%	0% − 40%	0% − 21%	0% − 21%	0% − 25%

**Fig. 2:**
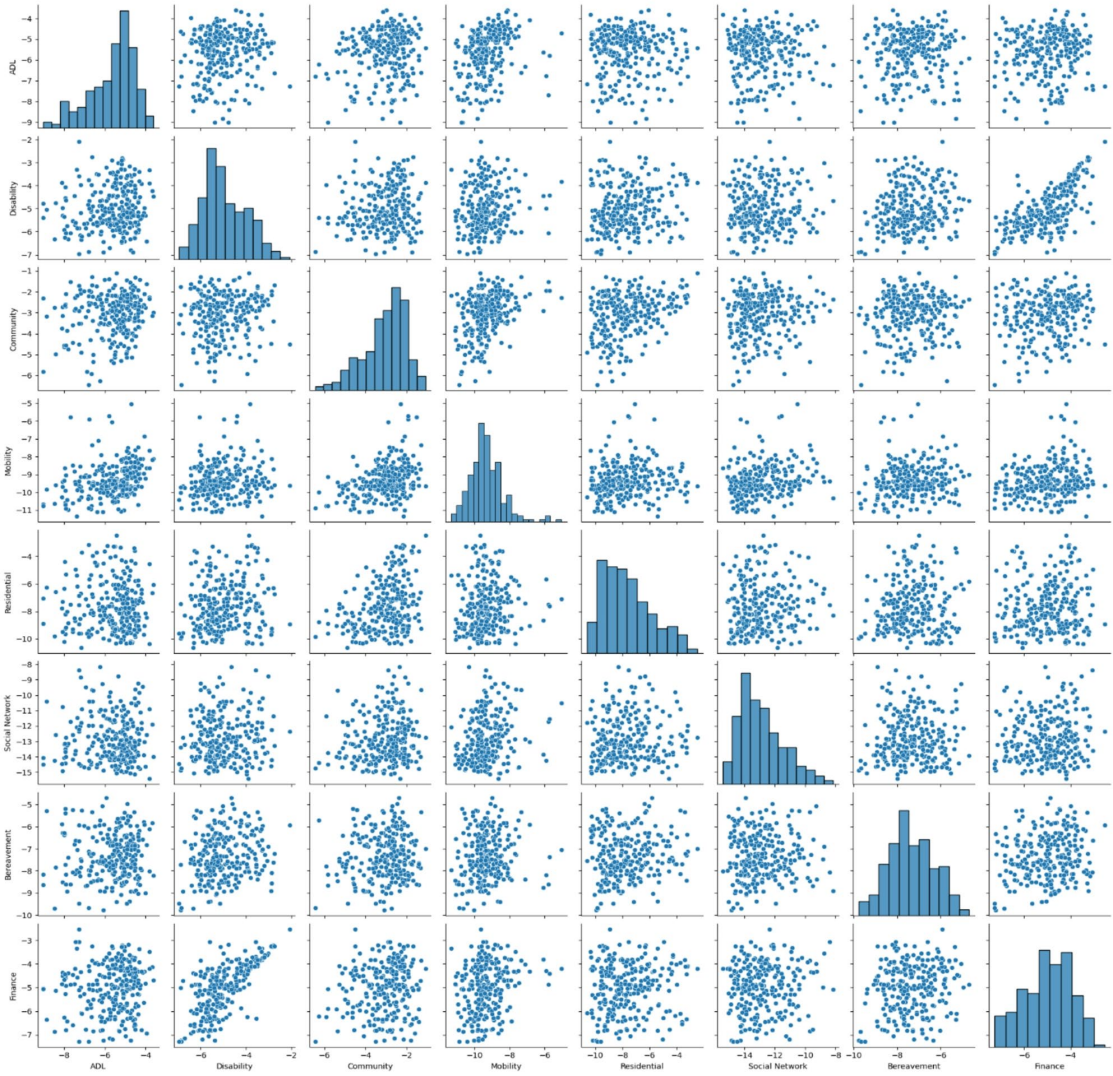
Pair-plot of the log-odds ratios of each Practice. Diagonal entries represent the histogram of log-odds for each SCN class, with off-diagonal entries representing scatter graphs of the joint log-odds for each SCN class pair

**Fig. 3:**
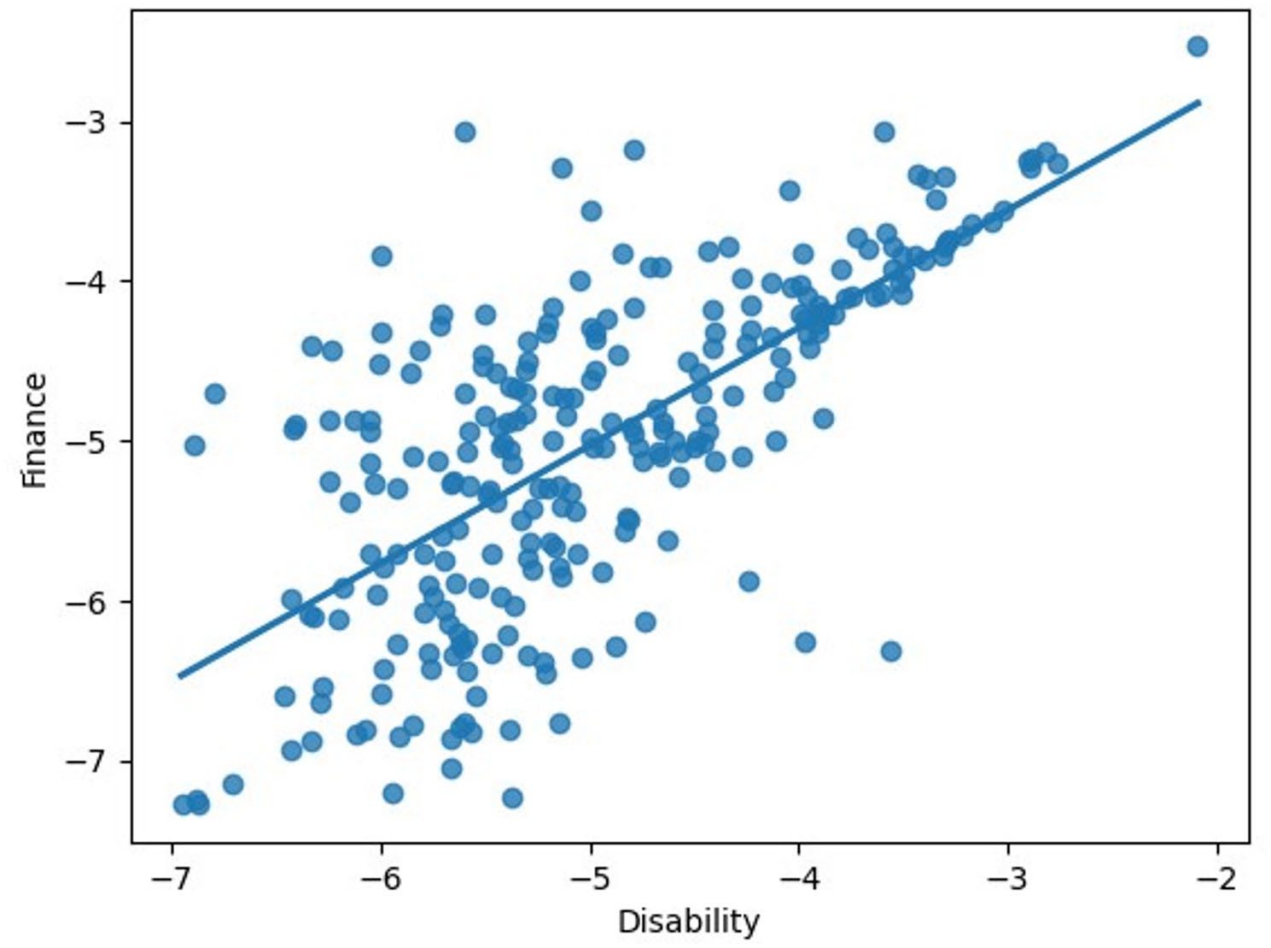
Scatter plot of log-odds of the finance and disability log-odds for each Practice. Linear regression line shown for visual representation of correlation

Moderate correlations were identified between reporting rates for two outcome pairs: mobility and activities of daily living (correlation 0.324), and disability and financial needs (correlation 0.675). We include a pair-plot of observed log-odds ratios for each practice and SCN in Fig. 2 and show the scatter plot for the disability and finances needs in Fig. 3. A full table of Spearman’s rank correlation between each pair of SCNs is given in Table 4.

**Table 4:** Spearman’s rank correlation between SCN classes at practice level

	ADL	Disability	Community	Mobility	Residential	Social Network	Bereavement	Finance
ADL	--	0.096	0.111	0.324	−0.019	−0.022	0.046	0.088
Disability	--	--	0.122	0.088	0.155	0.059	0.159	0.675
Community	--	--	--	0.340	0.315	0.121	0.054	0.147
Mobility	--	--	--	--	0.138	0.220	0.119	0.204
Residential	--	--	--	--	--	0.123	0.081	0.092
Social Network	--	--	--	--	--	--	0.033	0.086
Bereavement	--	--	--	--	--	--	--	0.151
Finance	--	--	--	--	--	--	--	--

These findings suggest that practices that reported higher rates of mobility difficulties or disability also tended to report increased difficulties with activities of daily living and financial challenges, respectively.

### Intra-class coefficients (ICCs)

Significant variability in the reporting of social care needs was observed between practices, with the intra-class coefficients (ICCs) for each model presented in Table 5. While there was some variation in the general recording of SCNs across practices, the Residential and Social Network SCN categories demonstrated considerably higher intra-class variation.

**Table 5:** Intraclass correlation coefficients for each social care need

Social care needs	Intraclass correlation coefficients
Any SCN	0.141
ADL	0.245
Disability	0.207
Community	0.239
Mobility	0.210
Residential	0.420
Social Network	0.348
Bereavement	0.228
Finance	0.222

### Social care needs reporting at patient level

The top five factors contributing most significantly to the reporting of each social care need (SCN) class are presented in Table 6. Across all outcomes, long-term conditions were the most influential factors. Chromosomal-related conditions, autism, dementia, and multiple sclerosis consistently ranked among the top five variables across all outcomes, indicating that patients with these conditions were more likely to have their social care needs reported.

**Table 6:** The five highest long-term condition odds ratios leading to a SCN report, broken down by individual SCNs, with the odds ratio and corresponding 95% confidence interval in parentheses

Variable order	ADL	Disability	Community	Mobility	Residential	Social Network	Bereavement	Finance
1st	Chromosomal (4.85 95% CI (3.71–6.34))	Chromosomal (55.06 95% CI (43.57–69.56))	Dementia (2.69 95% CI (2.56–2.82))	Chromosomal (20.45 95% CI (14.72–28.40))	Chromosomal (2.86 95% CI (1.72–4.77))	Chromosomal (7.89 95% CI (3.95–15.77))	Depression (1.63 95% CI (1.56–1.71))	MS (4.23 95% CI (3.58–5.00))
2nd	Hearing (4.75 95% CI (4.49–5.03))	Autism (17.39 95% CI (14.99–20.17))	MS (2.08 95% CI (1.87–2.30))	Autism (8.44 95% CI (6.45–11.04))	Dementia (2.85 95% CI (2.58–3.14))	Autism (6.26 95% CI (3.76–10.40))	Cystic (1.38 95% CI (0.74–2.56))	Pain (2.53 95% CI (2.36–2.72))
3rd	Autism (2.60 95% CI (2.13–3.19))	MS (3.38 95% CI (2.76–4.13))	Diabetes (1.85 95% CI (1.81–1.89))	MS (7.91 95% CI (6.60–9.48))	Autism (2.77 95% CI (2.02–3.79))	MS (2.85 95% CI (1.95–4.17))	Anxiety (1.26 95% CI (1.20–1.32))	HIV (2.08 95% CI (1.42–3.05))
4th	Menieres (1.90 95% CI (1.71–2.11))	Epilepsy (3.38 95% CI (3.11–3.67))	Pain (1.82 95% CI (1.75–1.90))	Parkinson (2.79 95% CI (2.32–3.35))	MS (2.54 95% CI (2.02–3.20))	Eating (2.80 95% CI (1.89–4.15))	Eating (1.21 95% CI (0.99–1.47))	Epilepsy (1.97 95% CI (1.81–2.15))
5th	MS (1.36 95% CI (1.13–1.62))	Visual (2.99 95% CI (2.56–3.48))	Autism (1.80 95% CI (1.59–2.05))	Paralysis (2.63 95% CI (2.34–2.95))	Visual (2.00 95% CI (1.69–2.37))	Schizophrenia (2.56 95% CI (1.95–3.36))	Addison (1.18 95% CI (0.75–1.86))	Osteoarthritis (1.94 95% CI (1.85–2.03))

Table 7 shows the odds ratios for the demographic exposures. Females were associated with higher rates of recording residential needs (OR 1.70 95% CI (1.61–1.80)) and needs resulting from bereavement (OR 2.16 95% CI (2.05–2.27)), relative to males. Patients in the most deprived IMD quintile (5th) had a higher likelihood of reporting disability needs (OR 2.27 95% CI (2.05–2.51)) and financial needs (OR 2.45 95% CI (2.24–2.67)) relative to the least-deprived decile (1st).

**Table 7:** Odds ratios for the demographic variables of each SCN model

Variable	ADL	Disability	Community	Mobility	Residential	Social Network	Bereavement	Finance
Age	1.03 (1.03–1.03)	0.98 (0.98–0.99)	1.01 (1.01–1.01)	1.05 (1.05–1.05)	1.02 (1.01–1.02)	1.09 (1.09–1.10)	1.03 (1.03–1.04)	0.99 (0.98–0.99)
Gender (Ref: Male)								
Female	0.81 (0.78–0.83)	0.90 (0.85–0.95)	1.22 (1.20–1.24)	1.09 (1.03–1.15)	1.70 (1.61–1.80)	1.13 (1.04–1.22)	2.16 (2.05–2.27)	0.94 (0.90–0.99)
Patient Index of Multiple Deprivation Quintile (Ref: 1st: Least Deprived)								
2nd	1.00 (0.96–1.05)	1.39 (1.26–1.54)	1.05 (1.02–1.08)	1.03 (0.94–1.11)	0.99 (0.91–1.08)	0.94 (0.84–1.05)	1.11 (1.04–1.19)	1.39 (1.27–1.52)
3rd	0.99 (0.94–1.04)	1.60 (1.45–1.77)	1.10 (1.07–1.13)	1.09 (1.00–1.19)	1.09 (1.00–1.19)	0.85 (0.76–0.96)	1.13 (1.05–1.21)	1.62 (1.48–1.76)
4th	1.02 (0.97–1.07)	1.97 (1.79–2.17)	1.10 (1.07–1.14)	1.15 (1.05–1.25)	1.10 (1.00–1.19)	0.94 (0.83–1.06)	1.16 (1.08–1.25)	1.98 (1.81–2.15)
5th (Most Deprived)	1.03 (0.97–1.09)	2.27 (2.05–2.51)	1.19 (1.15–1.23)	1.40 (1.28–1.52)	1.14 (1.03–1.25)	1.06 (0.93–1.21)	1.21 (1.11–1.31)	2.45 (2.24–2.67)
Ethnicity (Ref: White)								
Mixed	0.95 (0.76–1.19)	1.15 (0.86–1.52)	1.19 (1.06–1.33)	1.08 (0.76–1.53)	1.02 (0.67–1.54)	0.68 (0.34–1.37)	0.83 (0.58–1.20)	0.95 (0.72–1.25)
Asian	0.97 (0.88–1.07)	1.10 (0.95–1.28)	1.23 (1.17–1.30)	1.36 (1.18–1.56)	0.86 (0.69–1.07)	0.93 (0.75–1.16)	0.78 (0.66–0.91)	1.30 (1.15–1.46)
Black	0.74 (0.64–0.85)	1.01 (0.85–1.20)	1.20 (1.12–1.29)	1.00 (0.83–1.21)	0.85 (0.61–1.20)	1.17 (0.91–1.52)	0.95 (0.78–1.16)	1.14 (0.99–1.31)
Other	0.93 (0.81–1.07)	1.21 (1.00–1.45)	1.03 (0.96–1.11)	1.09 (0.88–1.36)	0.93 (0.70–1.24)	1.01 (0.75–1.35)	0.71 (0.56–0.90)	1.28 (1.10–1.50)

## Discussion

In this study, we examined the variability in the reporting of SCN amongst GP practices in England for individuals with multimorbidity. We found significant variability in the reporting of SCN across GP practices. Mobility and residential needs exhibited the widest variation in reporting. Moderate correlations were observed between some SCN categories, such as mobility and activities of daily living (ADL). Not all long-term conditions (LTCs) were equally likely to have SCNs reported; conditions like dementia and multiple sclerosis were more likely to have their SCNs reported. Additionally, demographic factors, including gender and socio-economic deprivation, influenced SCN documentation rates, with females and those from more deprived areas being more likely to have their needs reported [25].

One factor that may contribute to variability in SCN reporting are differences in clinical coding practices across GP practices. This heterogeneity in how healthcare professionals and individual practices record social determinants of health can be influenced by a range of factors, such as differences in training, familiarity with coding systems, and the perceived clinical relevance of social care and the social needs of patients [26–29]. Furthermore, some practices may prioritise the recording of SCNs as part of a holistic care approach to multimorbidity patients, whilst others may focus primarily on biomedical factors as the priority for primary care. It is also important to note that reporting of social care needs may not always come directly from the individual. For example, patients with advanced dementia or other neurodegenerative conditions may be unable to articulate their needs and instead rely on informal caregivers to convey them. In such cases, needs may be less visible within primary care records and therefore under-recorded.

We observed moderate correlations between several SCN categories, particularly mobility and ADLs. This finding is expected, as mobility limitations often impair an individual’s capacity to perform essential self-care tasks associated with ADLs, such as dressing, bathing, cooking and cleaning, as functional capability is a necessary prerequisite for these tasks [30]. This correlation may also result from health and social care services identifying and recording mobility impairments and ADL difficulties together, as both require similar interventions, such as home adaptations, assistive devices, and personal care support [31]. Consequently, individuals with mobility difficulties are more likely to be assessed for ADL limitations and vice-versa.

We found that SCN reporting was not consistent across all long-term conditions (LTCs), with conditions such as dementia and multiple sclerosis being more frequently documented. The higher reporting of SCNs for dementia and multiple sclerosis may be related to the significant functional and cognitive impairments associated with these LTCs, which increase dependency and require holistic care across a range of domains, including social care interventions [2, 32]. In contrast, other chronic conditions may not be as overtly associated with SCNs, possibly due to factors such as certain LTCs requiring a low level of social care intervention, clinician perceptions and prioritisation of care needs or because some patients have informal care support in place, reducing the need for formal social care. An additional limitation is that the documentation of a social care need does not necessarily indicate the severity of that need. The CPRD dataset records the presence of a SCN but not its complexity or intensity, and therefore, our analysis is unable to reflect the severity of need.

Additionally, demographic factors, particularly gender and socio-economic deprivation, influenced SCN documentation rates. We found that females and those from more deprived areas were more likely to have their needs reported. These higher documentation rates may reflect higher levels of care need and different healthcare behaviours in accessing care among these populations. It is well documented that women are more likely to access primary care services and present with care-related concerns, which could contribute to increased SCN documentation [33, 34]. Gender differences in caregiving within heteronormative relationships may also influence the identification of social care needs. For example, women are less likely to have a spouse provide care, in part because men have shorter life expectancies and fewer years in good health after 65 years, and in part due to differences in perceived caregiving capacity. This dynamic may contribute to greater identification of social care needs among women in primary care, although further research is needed to explore this in the context of multiple long-term conditions. Similarly, individuals living in socio-economically deprived locations are more likely to have higher levels of complex care needs, leading to more frequent presentations and use of healthcare services, resulting in higher rates of SCN reporting [15, 35].

### Comparison to existing literature

There is a limited evidence base in this field. To our knowledge, this is the first study to highlight significant variability in the documentation of social care needs across healthcare practices using electronic health records in a population-based sample. Previous studies have generally focused on specific practices or smaller datasets [25], and our findings provide new insights into the extent of variability in reporting social care needs at a national level. This study also aligns with other studies that have highlighted the underreporting of SCNs [36]. The underreporting of social care needs remains a key concern, as it may hinder the development of effective interventions for patients with complex care requirements, such as those with multimorbidity [13]. The findings of this study highlight a significant gap in the evidence base, demonstrating the need for future research to investigate the specific factors underlying the underreporting of SCN, especially in the context of primary care.

Our results also align with the literature, indicating that certain long-term conditions, particularly those with more visible or complex care requirements (e.g., dementia, multiple sclerosis), are more likely to be associated with social care needs reporting [19]. Furthermore, the influence of socio-economic status on reporting practices is well-documented, with individuals from more deprived socio-economic backgrounds often facing increased health disparities, including unmet social care needs [18]. This study extends these findings by demonstrating how variations in reporting practices may exacerbate these disparities.

### Strengths and limitations

This study has a number of strengths. It uses a large, population-based dataset, which provides a robust overview of SCN reporting across a wide range of GP practices in England. The use of real-world data from a national sample enhances the generalisability of the findings and underscores the relevance of the study to broader healthcare contexts. Additionally, the identification of specific long-term conditions and demographic factors associated with SCN reporting provides valuable insights into the areas where reporting practices may require improvement.

However, the study has several limitations; the observational nature of the data means that causality cannot be inferred from the associations observed. Second, whilst the dataset includes a large number of patients, there may be unmeasured confounders, such as variations in practice-specific protocols for SCN documentation or differences in healthcare professionals’ awareness and training regarding social care needs. The reliance on existing clinical records also means that missing or incomplete data could have influenced the findings, particularly concerning socio-economic or demographic variables. Further, the data we used does not consider the stage of disease progression, for example, if an individual has advanced dementia or other degenerative condition, they are likely to need additional care and support and therefore have a greater number of SCN. An additional limitation is that the documentation of a social care need does not necessarily indicate the severity of that need. The CPRD dataset records the presence of a SCN but not its complexity or intensity, and therefore, our analysis is unable to reflect the severity of need.

Finally, the study focused solely on GP practices recorded data (although linked to hospital records) and as a result, it does not account for variations in SCN reporting across other settings, such as social services or community-based services. It is important to note that our study measures reporting of SCN in primary care records, not the full population prevalence of SCN. Differences observed across demographic and clinical groups may therefore reflect a combination of both underlying need and patterns of documentation within GP practices.

### Implications for practice

The findings from this study have several important implications for practice. The observed variability in SCN reporting across General Practices highlights the need for standardised guidelines and training to improve the consistency of social care need documentation. General Practices may benefit from targeted interventions that promote the identification and documentation of social care needs, particularly for conditions that are less likely to be associated with formal social care assessments, such as those related to mobility or social networks. Training programmes for healthcare professionals could emphasise the importance of comprehensive social care assessments for patients with multimorbidity, ensuring that both medical and social factors are considered in care planning. Furthermore, the identification of particular demographic groups, such as females and individuals in deprived areas, being more likely to have their SCNs reported, could inform policies aimed at addressing health inequalities. Ensuring that SCNs are fully recognised and recorded is essential for facilitating integrated care, improving health outcomes, and enabling social prescribing. Ultimately, our findings suggest that improving the systematic documentation of social care needs can play a pivotal role in enhancing holistic care for patients with multimorbidity. By addressing the unmet social care needs of this population, healthcare providers can potentially reduce the burden of long-term conditions and promote better health trajectories, particularly for the most vulnerable individuals in society.

## Conclusions

Overall, the variability in SCN reporting likely reflects a combination of systemic, institutional, and variations in clinician recording practices [8, 37]. The introduction of standardised documentation practices and integration of social care assessments within EHRs could potentially mitigate inconsistencies in recording and ensure more effective and equitable identification of social care needs. Future research should explore methods and practices to improve the consistency of SCN reporting across primary care settings.

## Data Availability

This study used Clinical Practice Research Datalink (CPRD) anonymised data, which is subject to a full licence agreement that does not permit data sharing outside of the research team and therefore, is not publicly available. Data can be obtained by applying to CPRD ([enquiries@cprd.com](mailto: enquiries@cprd.com)) for any replication of the study. The READ and SNOMED codes used by this study are available from the corresponding author upon reasonable request and with the permission of the Clinical Practice Research Datalink.
